# Southern v. Thomas and Skyrme

**Published:** 1906-12-08

**Authors:** 


### The Hospital

A Journal of

The Medical Sciences and Hospital Administration.

Vol. XLI.—No. 1,055. SATURDAY, DECEMBER 8, 1906.

### SOUTHERN V. THOMAS AND SKYRME.

The King's Bench, from November 22 to December 3, has been the seat of an action brought by a mining engineer against Mr. Lynn Thomas, of Cardiff, and Mr. Skyrme to recover damages for alleged negligence in their treatment of the plaintiff. The patient had sustained an injury to the shoulder, which was thought to be a dislocation and a slight fracture of the upper end of the humerus, amounting, perhaps, to a mere chipping off of a piece of the bone. It proved, however, to be a fracture of the upper third of the humerus at the neck, which may have been associated with a dislocation; and the plaintiff sought to show this mistake amounted to negligence, as a consequence of which he lost the use of his arm. The case was tried before Mr. Justice Bigham and a special jury, and was somewhat remarkable on account of the amount of expert testimony produced on both sides, as surgeons were called and gave evidence from several of the hospitals in London to which medical schools are attached. The jury, after considering their verdict for two hours and a half, awarded the plaintiff £100 damages against both the defendants. The case had been tried previously at the Cardiff Assizes last December, when the jury failed to agree.

Many lessons are to be learnt from such a trial. It is well known that the less common fractures and injuries in the neighbourhood of joints are, by their results, extremely liable to give rise to great dissatisfaction, which may culminate, as in the present instance, in a damaging action for malpraxis. It is important, therefore, to take every means to obtain an accurate diagnosis at once and to explain fully to the patient the nature of the injury and the possible results, even though the accuracy of the diagnosis makes no material change in the treatment. Early recognition is imperative because the signs are soon masked by the swelling which is the usual accompaniment of such injuries—a swelling due in part to the escape of blood, in part to plugging of the veins, and in part to the inflammation following injury to the tissues. It is incumbent upon the surgeon, therefore, to obtain an *x*-ray photograph as quickly as possible after the accident, and it would be wisdom on his part to reserve his diagnosis until he has seen the negative. The taking of a skiagram was omitted in the present case with the good-natured intention of saving the patient the expense which it would have entailed; yet the judge, in his summing-up, referred to the absence of such a skiagram as a negative act of carelessness. The entire absence of even an elementary knowledge of pathology was plainly shown during the trial by questions as to whether the blood did not clot only in veins and not in extravasations and by the faith displayed as to the efficacy of the manipulation known as "setting a fracture." The omission of the latter act the presiding judge went so far as to say was the strength of the plaintiff's case, and from this it is clear that he believed if the fracture had been so set the two ends would have remained in apposition, and a useful arm would have been the result. It is well known to everyone who has to deal with fractures in the immediate neighbourhood of a large joint that the effect of setting a fracture is quite transient as regards the position of the fractured ends of the bones. It is impossible to keep the fragments in more than momentary apposition, and the real use of the manœuvre is to draw the lower end of the broken bone downwards, and thus to free it from any soft structures in which it may have become entangled or against which it is pressing unduly. A splint may afterwards be applied to steady the limb, but the everyday practice of Dr. Lucas-Championnière in Paris shows that absolutely satisfactory results are obtained by passive movement, and that our conservative methods are productive of harm rather than good. The result of this action therefore is to deter surgeons from availing themselves of other than routine practices, for if they use means not detailed in English text-books on surgery they may be blamed rather than applauded for their ingenuity.

The lessons taught by the trial to some of the medical witnesses are chiefly concerning the respect due to a court of law in this country. The conduct of most of the witnesses was so unexceptional that it made the slightest breach of decorum the more noticeable, and it is desirable that skilled witnesses should not relate their dreams, or perform, in open court, what counsel can describe as conjuring tricks. Nothing but sympathy can be extended to Mr. Lynn Thomas and to Mr. Skyrme for the anxiety through which they have passed, and in which they must continue awhile longer, for at the conclusion of the trial Mr. Colam, for the defendants, asked for a stay of execution. Some part of the heavy expense of so long a trial will doubtless be taken from them, as the Medical Defence Union was concerned in the trial at Cardiff; but nothing can make up for the actual wear and tear accompanying so prolonged a contest.

